# DAILY STRESS, DYADIC COPING, AND COGNITIVE FUNCTIONS IN MIDLIFE SAME- AND DIFFERENT-SEX MARRIAGES

**DOI:** 10.1093/geroni/igac059.2074

**Published:** 2022-12-20

**Authors:** Yiwen Wang

**Affiliations:** University of Texas at Austin, Austin, Texas, United States

## Abstract

Little is known about same-sex couples’ stress experience and cognitive performance at older ages as compared to different-sex couples. Sexual minority older adults may be at greater risk of cognitive impairment and dementia than their non-LGB counterparts due to life-long exposure to the discrimination and stigma associated with sexual minority status. In this study, I use daily dyadic longitudinal data from the Health and Relationships Project (HARP) to explore the patterns of daily cognitive functioning (memory failures and unconstructive repetitive thinking) among spouses in same-sex and different-sex marriages within the United States (N = 836 individuals, 419 couples). The primary focus of this study is to assess how daily stress—as reported by each spouse—is associated with cognitive function, and how these associations vary for women and men in same- and different-sex marriages. To examine how dyadic coping (the process through which couples manage stress together) matters, I also test whether collaborative dyadic coping buffers the health impact of daily stressors on cognitive function. Results suggest that same-sex couples experience more daily stress and have worse cognition compared to different-sex couples. While both respondent- and spouse-reported daily stress are negatively associated with cognitive function, the association between spouse-reported stress and cognition is stronger for women in same-sex marriages compared to men in different-sex marriages. Both men and women in same-sex marriages are more likely to cope with stress collaboratively than their counterparts in different-sex marriages, which helps buffer the negative consequences of daily stress on cognition.

